# Innovating fire safety with recombinant hydrophobic proteins for textile fire retardancy

**DOI:** 10.1111/1751-7915.14340

**Published:** 2023-09-25

**Authors:** Katie A. Gilmour, Thora H. Arnadottir, Paul James, Jane Scott, Yunhong Jiang, Martyn Dade‐Robertson, Meng Zhang

**Affiliations:** ^1^ Hub for Biotechnology in the Built Environment, Department of Applied Sciences Northumbria University at Newcastle Newcastle upon Tyne UK; ^2^ Hub for Biotechnology in the Built Environment, School of Architecture, Planning and Landscape Newcastle University Newcastle upon Tyne UK

## Abstract

Fire retardancy for textiles is important to prevent the rapid spread of fire and minimize damage to property and harm to human life. To infer fire‐resistance on textile materials such as cotton or nylon, chemical coatings are often used. These chemicals are usually toxic, and economically and environmentally unsustainable, however, some naturally produced protein‐based fire retardants could be an alternative. A biofilm protein from *Bacillus subtilis* (BslA) was identified and recombinantly expressed in *Escherichia coli* with a double cellulose binding domain. It was then applied to a range of natural and synthetic fabric materials. A flame retardancy test found that use of BslA reduced fire damage by up to 51% and would pass fire retardancy testing according to British standards. It is therefore a viable and sustainable alternative to current industrial fire‐retardant coatings.

## Graphical abstract

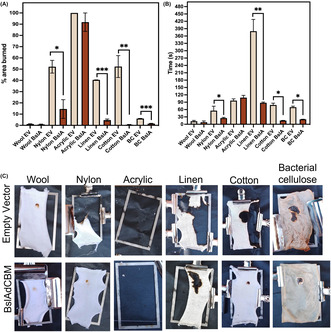

## INTRODUCTION

Combustible materials are present across the built environment including raw building materials such as wood or insulation materials, as well as textiles used for furnishing. A host of technological defences are implemented in homes and public spaces to safeguard from fire including smoke alarms and sprinkler systems and more specifically, the textiles themselves are treated to increase their fire retardancy.

Although some textile materials, including wool, are naturally fire‐resistant (Cardamone, 2013), the increased use of flammable materials such as synthetic hydrocarbon‐derived fibres including nylon and acrylic, necessitates a treatment to reduce the risk of fire spreading. Currently, chemicals, minerals and halogenated compounds are used to increase fire retardancy (Morgan & Gilman, 2013); and although cost‐effective, on combustion the products of these compounds have been found to be carcinogenic and increase corrosive gases which can remain in the environment long after the fire has been extinguished (Laoutid et al., 2009).

Previous work has identified casein, a protein commonly found in milk, as a possible green flame‐retardant coating (Alongi et al., 2014; Leong et al., 2021). Other naturally occurring proteins have also been investigated for similar properties, including fungal hydrophobins (Alongi et al., 2014). Here we investigate the potential of a bacterial hydrophobic protein, BslA which stands for biofilm surface layer protein A (Kobayashi & Iwano, 2012; Morris et al., 2017), to infer fire retardancy on synthetic and natural fibres. The limitation that has been identified with using proteins on textiles for the purposes of functionalization has been their lack of adhesion to the textile particularly following washing, however, by using an engineered biology approach to produce BslA with an adhesive domain, cellulose binding module (CBM), the bind efficiency and binding durability of the protein to cellulosic textiles post‐washing can be increased (Florea et al., 2016; Gilbert et al., 2021; Gilmour et al., 2023; Griffo et al., 2019). We demonstrated that a wide range of fabrics treated with our engineered proteins have better fire retardancy. These findings offer fresh perspectives for creating fire‐retardant molecules that are environmentally sustainable.

## EXPERIMENTAL PROCEDURES

### Expression of recombinant BslA in *E. coli*

Recombinant BslA and green fluorescent protein (GFP) with a double CBM (dCBM) were produced in *Escherichia coli* as described previously [7]. In brief, the pET28a containing corresponding sequences was transformed into *E. coli* BL21 (DE3). The protein was expressed in Luria–Bertani (LB) medium supplemented with 100 μg/mL kanamycin, at 30°C and 100 rpm for overnight. After harvesting, the cells were lysed through sonication in the buffer containing 100 mM Tris, 0.5 mM NaCl and 20 mM imidazole. This solution (cell free extract (CFE)) was used for material treatment.

### Bacterial cellulose non‐woven fabric production

The cellulose producing bacterium *Komagataeibacter xylinus* DSM 2325 was used to produce non‐woven fabric in Hestrin–Schramm (HS) medium. A starter culture was prepared as previously described (Gilmour et al., 2023) and pellicles were grown in 300 mL of HS media in Microboxes (160 mm × 160 mm, SacO_2_, Belgium). After 14 days growth, pellicles were harvested and washed in dH_2_O for 3 h, then overnight. The pellicles were then washed in 1 M NaOH for 16 h and finally in dH_2_O for 6 h. All wash steps were carried out at 20°C, 60 rpm. The pellicles were then dried on baking paper in a humidity chamber (Bambi compressor model VTS150D) with 15% humidity at 40°C.

### Textile/fabric preparation

Fabrics were knitted in a plain knit structure on a 12gg Shima Seiki SSR knitting machine. The fabrics were knitted from a range of natural and synthetic fibres from yarns supplied by Uppingham Yarns UK. The yarn count for acrylic, nylon (Rediver) and linen were 2/28 nm, the yarn count for merino wool was 2/30 nm and cotton was 3/46Ne (resultant 9/46Ne).

### Textile treatment and fire retardancy testing

All textile samples were cut to 80 mm × 160 mm and submerged in 50 mL of 5 mg/mL CFE solutions for 10 min. The samples were then dried flat at 20°C for 48 h before being mounted onto a metal frame measuring 80 mm × 160 mm and secured using metal bulldog clips. To quantify the proteins coated on the fabric samples, the left over CFEs were analysed using Bradford protein assay (Sigma, UK). In addition, to determine the action of the dCBM, the coating process was repeated in 50 mL of 5 mg/mL CFE solution of GFPdCBM. Fluorescence of the CFE solution before and after submersion of textiles was recorded, as well as the volume change to determine the amount of protein on each sample.

**FIGURE 1:**
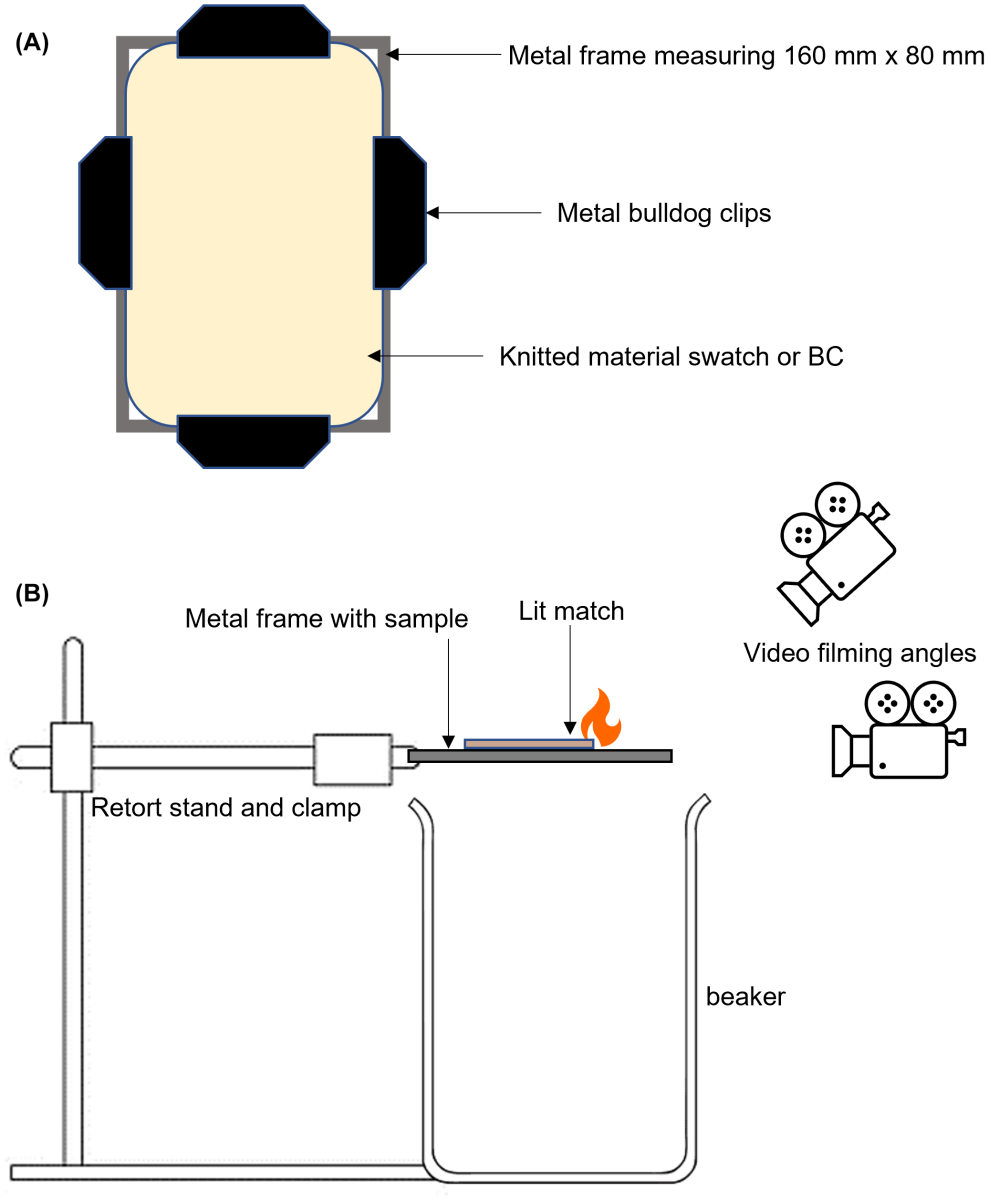
The adapted method for fire retardancy testing: (A) Textile samples clipped to a metal frame. (B) Fire retardancy set‐up and filming arrangement.

Fire retardancy tests were carried out according to a modified protocol of British standard (BS) BS EN 1021‐2. The frame (Figure 1A) was held by a laboratory clamp on a retort stand over a flame proof beaker outside. The temperature was 13°C and the humidity was 69%, in keeping with testing limits. As shown in Figure 1B, a lit match was then placed on the centre of the sample and the fire was recorded from two angles (Canon EOS M50 with Canon EF‐M 28 mm macro IS STM lens and Fujifilm X‐T2). ImageJ was then used to determine the burnt area of sample.

The textile samples treated using CFE of *E. coli* transformed with empty pET28a (EV) were controls and all the testing were performed in triplicates.

### Statistical analysis

Statistical analysis of results obtained from ImageJ analysis of the area burned of each textile sample was carried out by analysis of variance (ANOVA) in R (RStudio Team, 2020) with a sample size of three (*n* = 3). *p*‐values were obtained following normality checks, and post‐hoc Turkey tests were carried out, *p*‐values <0.05 were determined to be significant.

## RESULTS AND DISCUSSION

**FIGURE 2:**
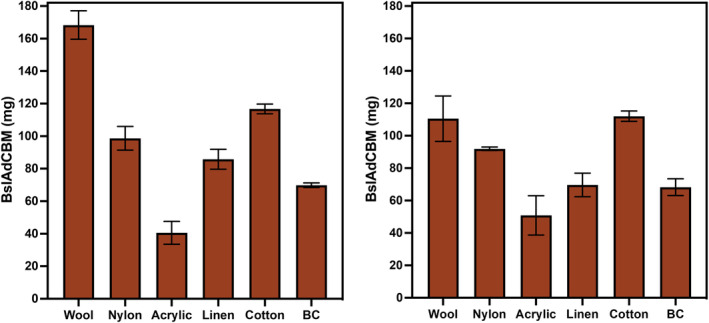
The amount of protein coated on each textile fabric: (A) Protein was quantified by Bradford protein assay; (B) Protein was quantified using fluorescence measurements. All the tests were conducted in the triplicates and error bars show standard error.

Experimental procedures were carried out to express recombinant BslA in *E. coli* and produce bacterial cellulose non‐woven fabric (Gilmour et al., 2023), which was then used to prepare various natural and synthetic textile samples. The textiles were treated with CFE solutions containing BslA with a double cellulose binding module (dCBM) and tested for fire retardancy according to a modified British standard protocol (Figure 1). To quantify the proteins coated on the fabric samples, the leftover CFEs were analysed using the Bradford protein assay (Sigma, UK) and fluorescence measurements. The trend in how much protein was on each sample followed the known trends of material absorbency (i.e. more absorbent textiles had a greater mass of protein), with a few exceptions (Figure 2). Bradford assay results (Figure 2A) were deemed unreliable due to interference from fibres and keratin in wool resulting in a large increase in protein concentration in the excess solution. Therefore, change in fluorescence measurements were used to determine protein concentration (Figure 2B).

Despite nylon having lower absorbency than linen, these samples had a higher mass of protein. We believe this is due to a coating present on the linen (size) which was added by the manufacturer. Wool was the most absorbent and therefore coated with the most protein (163 mg per sample) and acrylic, due to its naturally non‐absorbent properties had the least protein at 34 mg per sample. Bacterial cellulose (71 mg protein per sample) most closely resembles cotton (115 mg protein per sample), however, as a pellicle is more akin to a non‐woven material than a knit and was considerably thinner, it absorbed less CFE solution.

**FIGURE 3:**
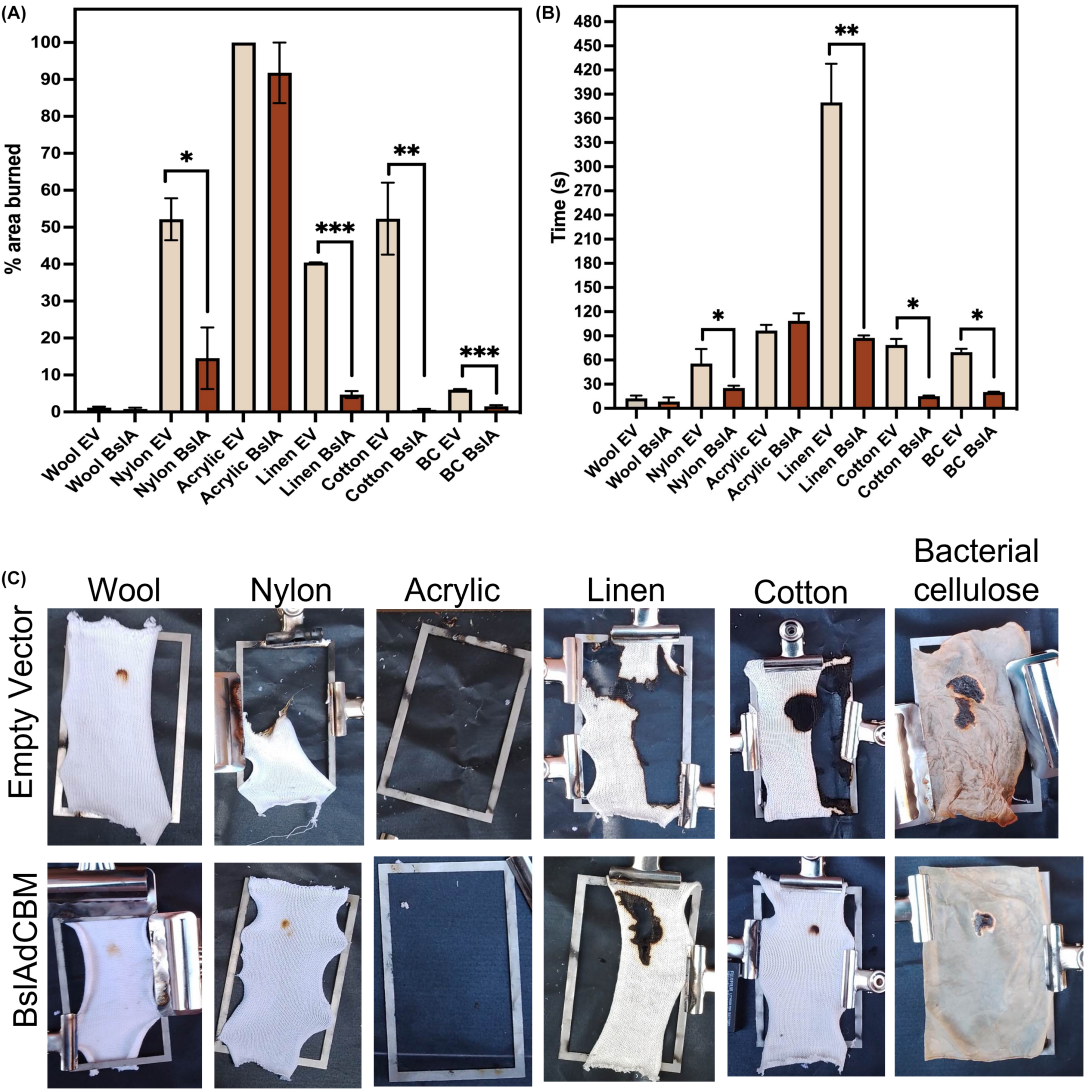
Fire retardancy tests: (A) The percentage of the area of textile burned following fire retardancy testing, error bars show standard error. (B) The time for the flame to go out, error bars show standard error. (C) Photographs of textile samples post fire retardancy testing.

Interestingly, when compared to fire retardancy testing a similar trend appears in that those with more protein had a smaller percentage area burned compared to those which had a lower mass of protein coated on the surface (Figure 3A). Images of the samples following fire retardancy tests are shown in Figure 3C, and when calculating the area of burned sample, those areas showing complete combustion or smouldering were included. Cotton, linen and BC are all composed primarily of cellulose fibres ((C_6_H_10_O_5_)n), the addition of a CBM increased affinity to the textile as reflected in the fluence measurement, and consequentially the difference in the burned areas in treated samples compared to control are highlight significant (*p*‐values of 0.006, <0.001, <0.001, respectively).

When compared to the EV control, neither wool nor acrylic were significantly more fire‐retardant when coated with BslAdCBM. Naturally, wool is flame‐resistant, as it is made of keratin and therefore has a high nitrogen content, additionally, as the fibres burn, they swell creating a less oxygen rich environment and preventing the spread of the fire (Cardamone, 2013). Whereas acrylic is substantially more flammable as it is composed of a synthetic monomer polyacrylonitrile ((CH_2_CHC)n) which once ignited will continue to burn and melt (Alongi et al., 2013). Additionally, this synthetic material has absorbency and as a result there was little protein on these samples. However, interestingly, when coated with BslAdCBM, nylon were significantly less burnt when compared to control too.

Additionally, the time for the flame to go out and smouldering to cease was decreased when samples were treated with BslAdCBM (Figure 3B). Similar to the area burned results, there was no significant difference between the wool and acrylic samples when compared to their respective controls. However, the burning time for all other samples was significantly reduced with *p*‐values of 0.0487, 0.0087, 0.0356 and 0.0127 for nylon, linen, cotton and BC, respectively.

Although similar to acrylic in that it is a synthetic material, nylon is composed of polyamides (most commonly (C_12_H_22_N_2_O_2_)n) and has a semi‐crystalline structure (Liu et al., 2007) whereas acrylic is significantly more amorphous (El‐Gabrie et al., 2014), this difference in property could account for the difference in affinity for protein. Studies have shown that certain CBMs have high affinity for synthetic hydrocarbon‐derived polymers and that this affinity is significantly increased if the fibre is of a high crystallinity, such as nylon when compared to acrylic (Rennison et al., 2023). Naturally, nylon melts slowly when ignited, and this melting acts as a barrier to the flame and normally the fire is not able to spread, unlike acrylic which more readily melts and drips, exposing further material to be burned (Alongi et al., 2013; Kundu et al., 2021).

Considering the criterion as described in BS5852: Part 1 and our results, wool would pass this test regardless of coating, however, nylon, linen, cotton and bacterial cellulose would only pass when coated with BslAdCBM, although the time to burn was <120 s in the majority of cases, the spread of the area burned was higher unless treated with BslAdCBM. When compared to other protein coatings, such as casein on cotton (Leong et al., 2021) our results show a decrease in burning time (15 s compared to 70 s) however, in this instance a vertical flame test was carried out with a lower concentration of protein coating which could explain the longer burn time.

Although toxicity testing was not carried out here, we believe the use of a natural protein would generate less toxic, or carcinogenic, fumes than the chemical and synthetic alternatives currently used (Morgan & Gilman, 2013). Not only does this pose a benefit to human health, but also to the environment in that less long‐lasting pollution would be generated.

## CONCLUDING REMARKS

The utilization of recombinant hydrophobic protein along with an adhesive domain (CBM) offers a promising and environmentally‐friendly alternative to conventional fire‐retardant coatings. We demonstrated that this method is not limited to cellulosic textiles but also holds the potential for synthetic textile materials. Furthermore, the use of an engineered biological approach allows for alteration of the binding protein for different textile needs and suggests further exploration of bacterial hydrophobins (or manipulation of the BslA used here) could enhance the protective quality. This process could thereby lead to the development of greener and more sustainable fire retardants in the future.

## AUTHOR CONTRIBUTIONS

**Katie A. Gilmour:** Conceptualization (supporting); data curation (lead); formal analysis (lead); investigation (lead); methodology (equal); validation (lead); visualization (lead); writing – original draft (lead). **Thora H. Arnadottir:** Methodology (supporting); visualization (supporting). **Jane Scott:** Funding acquisition (supporting); methodology (supporting); writing – original draft (supporting). **Paul James:** Funding acquisition (supporting); methodology (supporting); writing – review and editing (supporting). **Yunhong Jiang:** Funding acquisition (supporting); writing – review and editing (supporting). **Martyn Dade‐Robertson:** Conceptualization (supporting); funding acquisition (supporting); supervision (supporting); writing – review and editing (supporting). **Meng Zhang:** Conceptualization (lead); funding acquisition (lead); methodology (supporting); project administration (lead); resources (lead); supervision (lead); writing – original draft (supporting); writing – review and editing (lead).

## FUNDING INFORMATION

This work was funded by the Research England E3 scheme (2019), Engineering and Physical Sciences Research Council (EP/V050710/1) and Biotechnology and Biological Sciences Research Council (BB/X011402/1).

## Data Availability

All data supporting this study are provided in full in the ‘Results’ section of this paper. [Correction added on 30 September 2023, after first online publication: the Data Availability Statement section has been added in this version.]
